# GhMPK17, a Cotton Mitogen-Activated Protein Kinase, Is Involved in Plant Response to High Salinity and Osmotic Stresses and ABA Signaling

**DOI:** 10.1371/journal.pone.0095642

**Published:** 2014-04-17

**Authors:** Jie Zhang, Dan Zou, Yang Li, Xiang Sun, Na-Na Wang, Si-Ying Gong, Yong Zheng, Xue-Bao Li

**Affiliations:** Hubei Key Laboratory of Genetic Regulation and Integrative Biology, School of Life Sciences, Central China Normal University, Wuhan, China; Wuhan University, China

## Abstract

Mitogen-activated protein kinase (MAPK) cascades play pivotal roles in mediating biotic and abiotic stress responses. Cotton (*Gossypium hirsutum*) is the most important textile crop in the world, and often encounters abiotic stress during its growth seasons. In this study, a gene encoding a mitogen-activated protein kinase (MAPK) was isolated from cotton, and designated as *GhMPK17*. The open reading frame (ORF) of *GhMPK17* gene is 1494 bp in length and encodes a protein with 497 amino acids. Quantitative RT-PCR analysis indicated that *GhMPK17* expression was up-regulated in cotton under NaCl, mannitol and ABA treatments. The transgenic *Arabidopsis* plants expressing *GhMPK17* gene showed higher seed germination, root elongation and cotyledon greening/expansion rates than those of the wild type on MS medium containing NaCl, mannitol and exogenous ABA, suggesting that overexpression of *GhMPK17* in *Arabidopsis* increased plant ABA-insensitivity, and enhanced plant tolerance to salt and osmotic stresses. Furthermore, overexpression of *GhMPK17* in *Arabidopsis* reduced H_2_O_2_ level and altered expression of ABA- and abiotic stress-related genes in the transgenic plants. Collectively, these data suggested that *GhMPK17* gene may be involved in plant response to high salinity and osmotic stresses and ABA signaling.

## Introduction

Plant growth is significantly affected by a range of environmental stresses, such as drought, high salinity, pathogen infection, cold and freezing. Higher plants are sessile organisms that can not move to escape diverse unfavorable environmental factors. In order to survive, plants have developed a great ability to adapt the changes in the environment. They are equipped with sophisticated mechanisms, such as the perception and transduction of stress stimuli. The mitogen-activated protein kinase (MAPK) signaling pathway is an elaborate and intricate complex that are highly conserved in eukaryotes and play important roles in response to various biotic and abiotic stresses [1], [2]. Each mitogen-activated protein kinase (MAPK) cascade is known to be one of the major phosphorylation pathways which are composed of three kinases, MAPK, MAPK kinase (MAPKK), MAPKK kinase (MAPKKK) and function downstream of sensors/receptors to regulate cellular responses. A highly conserved MAPK cascade consists of three steps: MAPKs are activated through phosphorylation on two threonine and tyrosine residues by upstream MAPKK, which are in turn phosphorylated on two serine/threonine residues by MAPKKK [3], [4]. Then activated MAPKs regulate the activities of diverse targets including transcription factors [5], cytoskeletal proteins [6], and other protein kinases [7].

Genome sequencing and annotation of *Arabidopsis* revealed the existence of 20 MAPKs, 10 MAPKKs and 80 MAPKKKs [8]. In addition, a similar repertoire of genes have been found in other plants, such as 17 MAPKs in rice [9], and 21 in poplar [10]. In plants, based on the phylogenetic analysis of amino acid sequences and phosphorylation motifs, MAPKs can be divided into two subtypes: one with a TEY amino acid motif and the other containing a TDY motif. The TEY subtype can be classified into three groups: A, B and C, while the TDY subtype forms group D. In the group D, MAPKs have a long C-terminal extension domain in relation to groups A, B and C, which serves as a docking site for MAPKKs, phosphatases and protein substrates [11], [12]. MAPKs in groups A and B are mostly involved in response to biotic, abiotic stresses and hormones [13]–[16]. MPK3, MPK6 and its apparent orthologs in other species, such as *Arabidopsis* MPK3 and MPK6 (belonging to group A), alfalfa SIMK and SAMK, and tomato LeMPK1/2 and LeMPK3 are all involved in defense-related signal transduction [17]. In *Arabidopsis*, group B MPK4 is involved in osmotic stress response pathways activated by hypoosmolarity [18]. The available information of group-C MAPKs is relatively limited, and it seems that part of them play a role in cell cycle regulation and environmental stress responses [19]. Microarray analysis detected circadian-rhythm-regulated expression of MPK7 [20]. Some members of group D MAPKs may be involved in pathogen infection, mechanical wounding processes and some may play roles in both biotic and abiotic stress responses. Two reported group D genes, *OsBWMK1* and *MsTDY1*, are induced by blast fungus and wounding [21]–[23].

Cotton (*Gossypium hirsutum*) is an important fiber crop in the world. However, its growth and yield are severely impacted upon by various biotic and abiotic stresses. Previously, *GhMPK2*, a group C MAPK gene was identified in cotton. The expression of *GhMPK2* is strongly induced by salinity, dehydration and ABA [24]. Here, a group D mitogen-activated protein kinase gene, *GhMPK17* was identified in cotton. The *GhMPK17* gene is preferentially expressed in anthers and cotyledons, and is also induced by salt, osmosis and ABA. Overexpression of *GhMPK17* in *Arabidopsis* enhanced plant tolerance to salt and osmotic stresses, suggesting that *GhMPK17* gene may be involved in cotton multiple abiotic tolerance.

## Materials and Methods

### Cotton Materials

Cotton (*Gossypium hirsutum*, cv. Xuzhou 142) seeds were surface-sterilized with 70% (v/v) ethanol for 1 min and 10% (v/v) H_2_O_2_ for 2 h, and then were washed with sterile water. The sterilized seeds were germinated on half strength of MS medium under a 16 h light/8 h dark cycle at 25°C for 5 days. Roots, cotyledons, and hypocotyls were cut from these sterile seedlings. Other tissues were derived from cotton plants grown in field for RNA extraction.

For ABA (abscisic acid), salt and osmotic treatments, the sterilized cotton seeds were germinated on MS medium under a 16 h light/8 h dark cycle at 25°C for 6 days, and then seedlings were transferred on MS agar medium containing 100 µM ABA, 200 mM NaCl, or 200 mM manntiol. The treated cotyledons and controls were harvested for RNA isolation.

### Isolation of *GhMPK17* Full-length cDNA and Gene

A cotton seedling cDNA library was constructed as described previously [25]. More than 4000 cDNA clones were randomly selected from the cDNA library for sequencing. One clone containing the full-length *GhMPK17* sequence was identified from these cDNAs. Subsequently, the full-length *GhMPK17* gene was amplified from cotton genome by PCR, using the proofreading *Pfu* DNA polymerase and gene-specific primers that were designed according to *GhMPK17* cDNA sequence.

### DNA and Protein Sequence Analysis

Nucleotide and amino acid sequences were analyzed using DNAstar (DNAstar Inc., USA). The peptide sequences were aligned with the ClustalW program (http://www.ebi.ac.uk). The conserved domains of the GhMPK17 were confirmed at the National Center for Biotechnology Information (NCBI) (http://blast.ncbi.nlm.nih.gov/Blast.cgi). Identification of protein domains and significant sites was performed with Motifscan (http://myhits.isb-sib.ch/cgi-bin/motif_scan). The phylogenetic tree was constructed by the NJ (Neighbor- Joining) method using MEGA5. A bootstrap analysis with 1000 replicates was performed to assess the statistical reliability of the tree topology.

### Quantitative RT-PCR Analysis

Expression of *GhMPK17* gene in cotton under normal conditions and under NaCl, mannitol and ABA treatments, and the expression of ABA- and stress-responsive genes in *Arabidopsis* were analyzed by real-time quantitative reverse transcription polymerase chain reaction (RT-PCR) using the fluorescent intercalating dye SYBR Green (Bio-Rad, USA) by the method described earlier [26]. A cotton ubiquitin gene (*GhUBI1*, GenBank accession number: EU604080) was used as a quantitative control in RT-PCR reactions. The primer sequences were as follows: *GhMPK17* forward 5′-GTTGCAAGCATCCGTGGAACCAGAAT-3′ and reverse 5′-TAAGACAGATTAAGAACCTCCAGAGG-3′; *GhUBI1* forward 5′-CTGAATCTTCGCTTTCACGTTATC-3′ and reverse 5′-GGGATGCAAATCTTCGTGAAAAC-3′.

Expression of ABA- and stress-responsive genes in *Arabidopsis* was assayed by quantitative RT-PCR described as the above, using gene-specific primers: *AtABI1* (At4g26080) forward 5′-AGATGGCAAGGAAGCGGATT-3′, reverse 5′-CAACCACCACCACACTTATG-3′; *AtABF4* (At3g19290) forward 5′-AACAACTTAGGAGGTGGTGGTCAT-3′, reverse 5′-TGTAGCAGCTGGCGCAGAAGTCAT-3′; *AtSOS2* (At5g35410) forward 5′-GGCTTGAAGAAAGTGAGTCTCG-3′, reverse 5′-GCTACATAGTTCGGAGTTCCACA-3′; *AtRAB18* (At5g66400) forward 5′-AGATGGCAAGGAAGCGGATT-3′, reverse 5′-CTTCTTCTCGTGGTGCTCAC-3′. Expression level of *AtACTIN2* was monitored with forward 5′-GAAATCACAGCACTTGCACC-3′ and reverse 5′-AAGCCTTTGATCTTGAGAGC-3′ primers to serve as internal control. The RNA samples were extracted from 2-week-old seedlings treated with or without 50 µM ABA, 100 mM NaCl and 100 mM mannitol for 6 h. Then total RNAs were reversely transcribed into cDNAs which were used as templates in quantitative PCR reactions.

For all the above quantitative real-time PCR analysis, the assays were repeated three times along with three independent repetitions of the biological experiments, and means of three biological experiments were calculated for estimating gene expression levels.

### Subcellular Location of GhMPK17 Protein

To construct *GhMPK17::eGFP* vector, the coding sequence (without stop codon) of *GhMPK17* was cloned into pBI121 vector which had eGFP sequence at *Xba* I/*Bam*H I sites, using primers P1 (5′-GGG TCTAGAATGTTTGGCAAAGACTTTTT-3′) and P2 (5′- CTTGGATCCAGCGACCAGTTTCTGCAACT-3′). Then the prepared vector was transferred into *Agrobacterium tumefaciens* strain LBA4404. The cultures were diluted to OD600 = 1 and infiltrated into the abaxial side of young tobacco (*Nicotiana tabacum*) leaves. For inducing transient expression of *GhMPK17::eGFP* fusion gene, tobacco plants were cultured for three days after infiltration. Then, the epithelial tissue of the tobacco leaves were observed under a SP5 Meta confocal laser microscope (Leica, Germany) with a filter set for GFP fluorescence (488 nm for excitation and 506–538 nm for emission). The digital images were taken and process by SP5 software (Leica, Germany).

### Construction of Overexpression Vector and *Arabidopsis* Transformation

The coding sequence of *GhMPK17* gene was amplified by PCR using proof-reading *pfu* DNA polymerase and cloned into pBI121 vector at *Bam*H I and *Sac* I sites, replacing *GUS* gene to generate chimeric CaMV (cauliflower mosaic virus) 35Sp:GhMPK17 construct. The construct was introduced into *Arabidopsis* by the floral dip method. Transformed seeds were selected on MS medium containing 50 mg/L kanamycin. Homozygous lines of T3 and T4 generations were used for phenotypic analysis of transgenic plants.

### Assay of Seed Germination and Cotyledon Greening/expansion

About 50 surface-sterilized seeds from each transgenic *Arabidopsis* line and wild type, respectively, were sowed on MS plates supplemented with or without different concentration of mannitol, ABA or NaCl, and placed at 4°C for 4 days, then moved to a growth room under a photoperiod of 16 h light/8 h dark at 22°C. The rate of seed germination (root emergence) was evaluated every day, and the number of seedlings whose cotyledons was green was counted after 10 days. Each experiment was repeated three times at least with identical results.

### Assay of Primary Root Elongation and Lateral Root Length

For assaying primary root elongation, 7-day-old transgenic *Arabidopsis* seedlings and wild type were transferred and cultured on MS medium supplemented with different concentration of mannitol, ABA or NaCl for 7 days. Then, the elongation of roots (final root length minus original root length) was measured and calculated. All experiments were repeated at least three times.

For assaying lateral root length, 7-day-old transgenic *Arabidopsis* seedlings and wild type were transferred and cultured on MS medium supplemented with 100 mM mannitol for 7 days. Then, the quantity and length of lateral roots of wild type and *GhMPK17* transgenic lines were measured and calculated. According to the data measured, lateral roots were artificially divided into eight groups. The percentage of each group occupied in all lateral roots of each transgenic line was calculated, using the wild type as a control. All experiments were repeated at least three times.

### Histochemical Detection of H_2_O_2_

The substrate 3,3′-diaminobenzidine (DAB) was used to visually detect H_2_O_2_ in plants according to the method described previously [26]. Briefly, one-week-old *Arabidopsis* seedlings were treated with or without 50 µM ABA, 100 mM NaCl and 100 mM mannitol for 6 h. Then, the samples were infiltrated in 1 mg/mL DAB solution for 6 h, followed by the treatment with 70% ethanol to remove chlorophyll. The seedlings were examined and photographed.

## Results

### Characterization of *GhMPK17*

**Figure 1:**
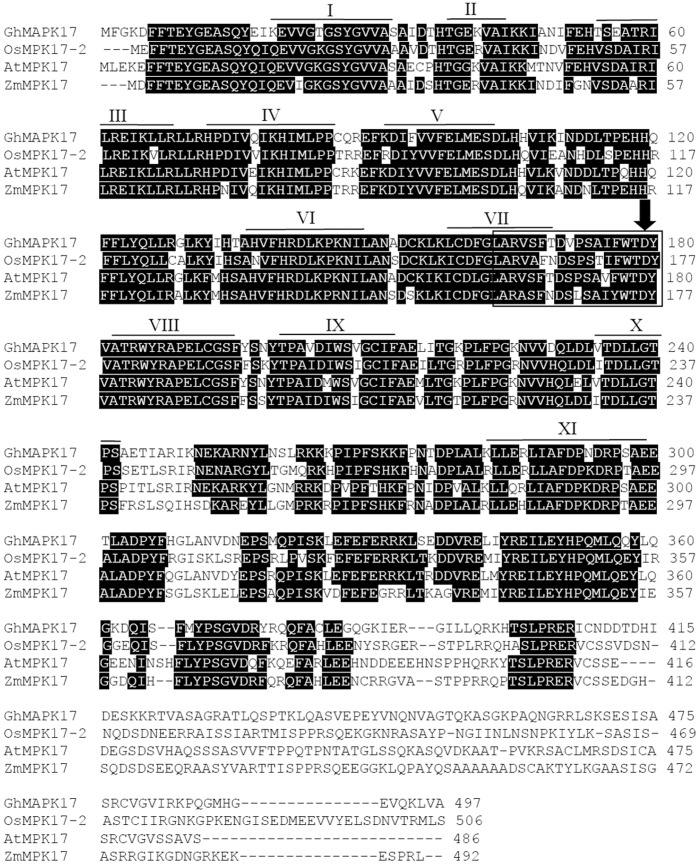
Characterization of GhMPK17 protein. Alignment of the deduced amino acid sequence of GhMPK17 with MAPKs from other plant species: AtMPK17 (NM001035863), ZmMPK17 (NM001154688.1), OsMPK17-2 (DQ826423.2). Protein kinase subdomains are shown by lines with numerals (I – XI), activation-loop motifs are boxed, and the conserved motif TDY is indicated by arrows.

**Figure 2:**
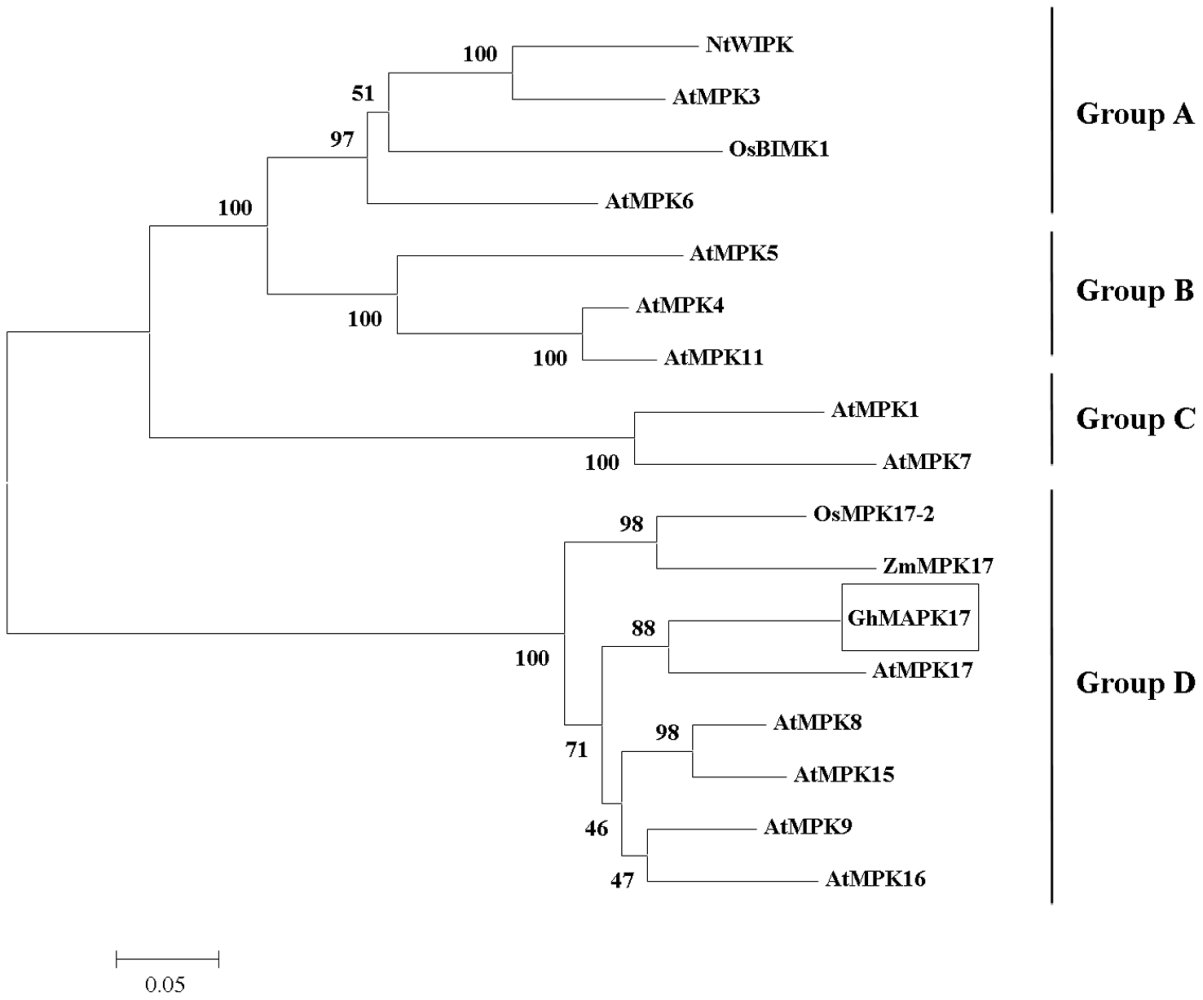
Phylogenetic relationship of GhMPK17 and other plant MAPK proteins. All 17 MAPK proteins can be divided into four groups (A, B, C, and D) based on their sequence similarities. Numbers above or below branches indicate bootstrap values from 1000 replicates. The plant MAPK proteins used for the phylogenetic tree are AtMPK1(NP172492), AtMPK3((NP190150), AtMPK4((NP192046), AtMPK5((NP567378), AtMPK6(NP181907), AtMPK7(NP179409), AtMPK8(NP173253), AtMPK9(NP566595), AtMPK11(NP001117210), AtMPK12(NP182131), AtMPK13(NP172266), AtMPK15(NP565070), AtMPK16(NP197402), AtMPK17(NP001030941), OsBIMK1(AAK01710.1), OsBIMK2(AAS18417), NtWIPK(BAA09600).

In order to identify genes involved in cotton stress response pathways, we randomly sequenced over 4000 cDNA clones from a cotton seedling cDNA library. Among these clones, one is a putative *MAPK* gene (cDNA) (designated as *GhMPK17*, GenBank accession number: KJ192192) that shares high similarity with other plant MAPKs (such as *AtMPK17*) in group D. The open reading frame (ORF) of *GhMPK17* is 1494 bp in length, and encodes a protein of 497 amino acids. Multi-alignment analysis revealed that GhMPK17 is highly related to other group D MAPKs. As shown in Fig. 1, GhMPK17 and other group D MAPKs shared the same 11 conserved subdomains, an activation loop, a C-loop, a P-loop, a phosphorylation motif TDY between subdomains VII and VIII. Meanwhile, GhMPK17 and its homologs, OsMPK17-2 (DQ826423.2), AtMPK17 (NM001035863), ZmMPK17 (NM001035863) had an extension C-terminal region relative to groups A, B and C. This C-terminal region showed a high level of variation. Based on the amino acid sequences of GhMPK17 and other plant MAPKs, phylogenetic analysis indicated that GhMPK17 belongs to group D (Fig. 2).

### Expression of *GhMPK17* in Cotton is Induced by Salt, Mannitol and ABA

**Figure 3:**
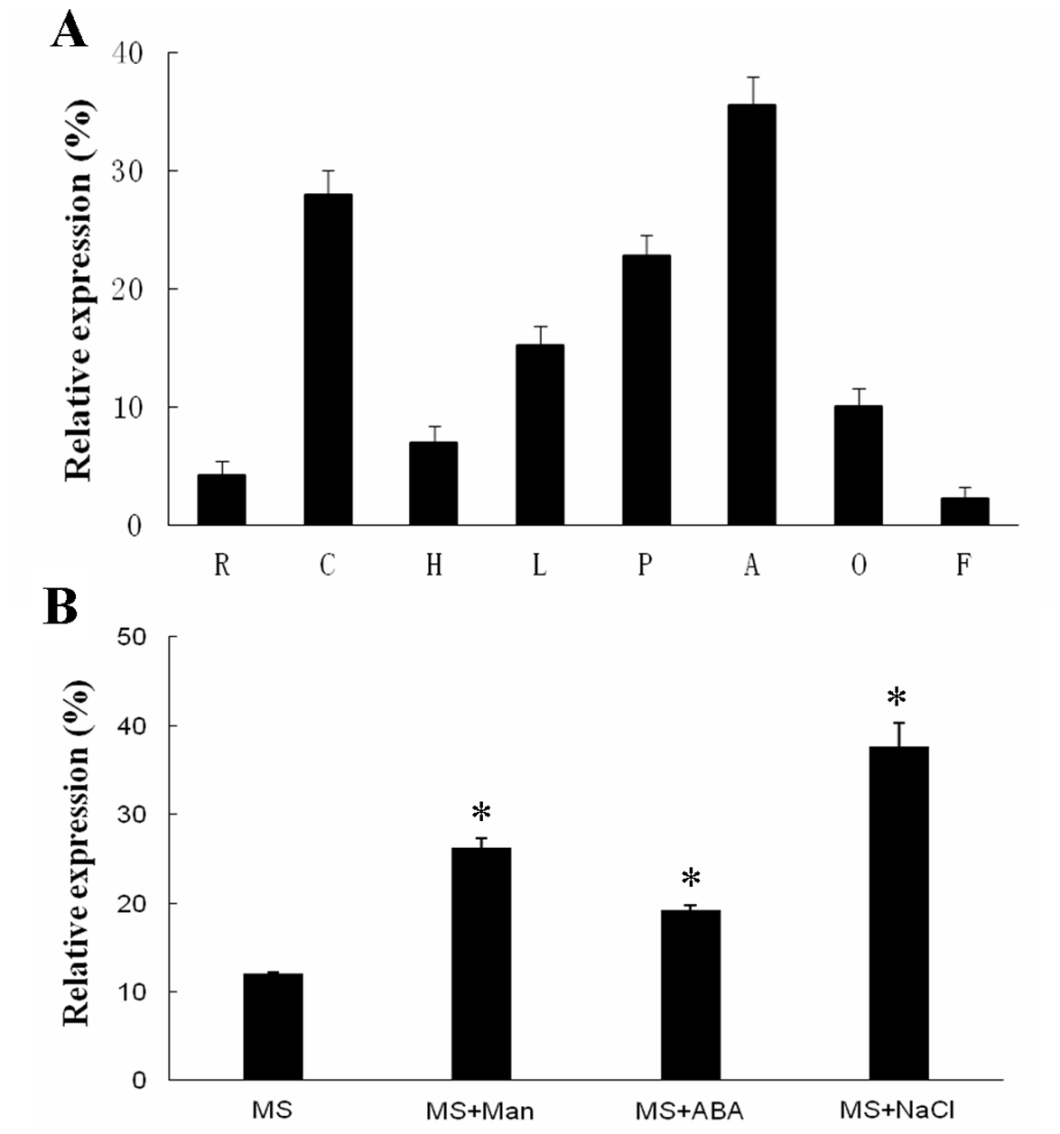
Quantitative RT-PCR analysis of GhMPK17 expression in cotton under normal conditions and different stresses. (**A**) Quantitative RT-PCR analysis of *GhMPK17* expression in cotton tissues. Total RNA was isolated from roots (R), hypocotyls (H), cotyledons (C), leaf (L), anthers (A), petals (P), ovules (O), and fibers (F). (**B**) Quantitative RT-PCR analysis of *GhMPK17* expression in cotton under different stresses. Total RNA was isolated from 6-day-old cotyledons treated with 100 µM abscisic acid (ABA), 200 mM NaCl and 200 mM manntiol (Man), respectively, using the untreated 6-day-old cotyledons as control (MS). Relative value of the expression of *GhMPK17* in cotton was shown as percentage of *GhUBI1* expression activity. Mean values and standard errors (bar) were shown from three independent experiments. *, P<0.05.

To determine the expression profile of *GhMPK17* gene in cotton, total RNA was isolated from different tissues of cotton. Expression levels of *GhMPK17* in cotton different organs or tissues were quantified by real-time quantitative RT-PCR (Fig. 3A). The results showed that *GhMPK17* was expressed at relatively high levels in both anthers and cotyledons, and at moderate levels in petals and leaves, whereas at relatively low levels in roots, hypocotyls, ovules and fibers.

It has been supposed that some members of MAPK group D are involved in response to abiotic stress and abscisic acid (ABA) treatment [27], [28]. To study whether expression of the isolated *GhMPK17* gene is regulated by abiotic stress and ABA, cotton seedlings were subjected to NaCl, mannitol and ABA treatments. As shown in Fig. 3B, *GhMPK17* expression in cotyledons was up-regulated by NaCl, mannitol and ABA treatments, suggesting that *GhMPK17* gene may be involved in plant response to abiotic stress and ABA signaling.

### Subcellular Localization of GhMPK17

**Figure 4:**
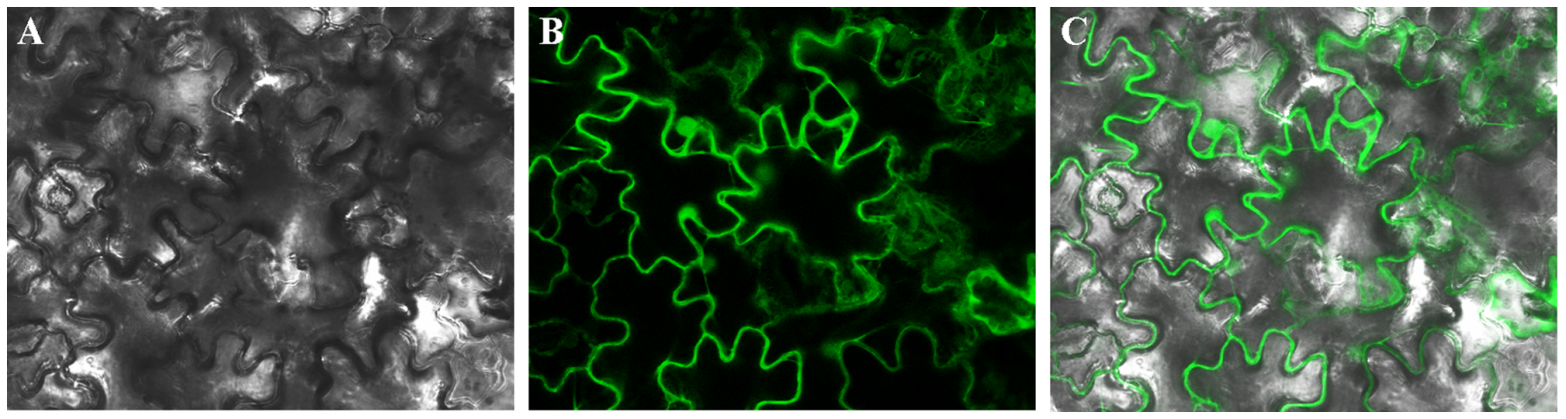
Subcellular localization of GhMPK17 protein. *eGFP* gene fused to C-terminal of *GhMPK17* gene was expressed in epidermal cells of transformed tobacco leaves. GFP fluorescence signals were mainly detected in cytoplasm of cells harboring the GhMPK17-eGFP reporter construct. Transmission image (A), GFP-derived fluorescence (B) and fluorescence superimposed over the transmission image (C) are shown.

It has been reported that MAPKs can phosphorylate target proteins in both the cytoplasm and nucleus, and the subcellular localization of MAPKs is extraordinary correlated with the resulting cellular response [29]. To reveal the intracellular localization of GhMPK17 protein, an *eGFP* reporter gene was fused to *GhMPK17* under the control of the cauliflower mosaic virus (CaMV) 35 S promoter. This 35 S:GhMPK17:eGFP construct was introduced into tobacco (*Nicotiana tabacum*) epidermal cells by agroinfiltration (see Methods). After a subculture of 48 hours, the GFP fluorescence was observed by confocal microscopy. As shown in Fig. 4, the GFP fluorescence signals were detected in cytoplasm of the transformed tobacco cells, suggesting that GhMPK17 protein may localize in the cytoplasm of cells for performing its function in cotton.

### GhMPK17 is Involved in ABA Signaling in the Transgenic *Arabidopsis* Seedlings

**Figure 5:**
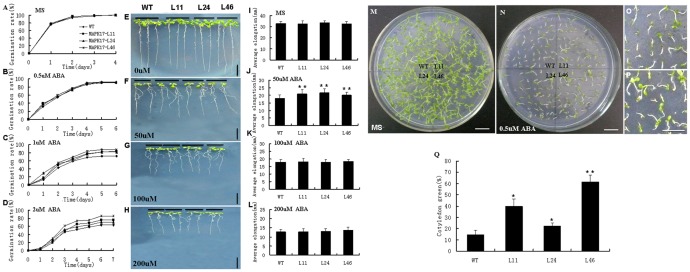
Assays of seed germination and root growth of transgenic Arabidopsis expressing *GhMPK17* under abscisic acid (ABA) treatment. (**A** to **D**) Seed germination rate of *GhMPK17* overexpression transgenic lines and wild type on MS medium containing different concentrations of ABA. Each curve represents an average of three replicates. (**E** to **H**) Phenotype of wild type and *GhMPK17* overexpression seedlings under ABA treatment. Seeds were germinated on MS medium under normal condition for 7 days and then the seedlings were transferred and cultured on MS medium supplemented with 0, 50, 100 and 200 µM ABA for 7 days. Each experiment was repeated at least three times with identical results. Bars = 1 cm. (**I** to **L**) Statistical analysis of root elongation of wild type and *GhMPK17* overexpression plants. Seeds were germinated on MS medium under normal condition for 7 days, and then the seedlings were transferred and cultured on MS medium with 0, 50, 100 and 200 µM ABA for 7 days. Data were shown from three independent experiments. (**M** and **N**): Post-germination seedling establishment analysis of *GhMPK17* overexpressing plants. (**M**) Seedlings of wild type and *GhMPK17* transgenic lines grew on MS medium without ABA for 10 days. (**N**) Seedlings of wild type and *GhMPK17* transgenic lines grew on MS medium with 0.5 µM ABA for 10 days. (**O** and **P**) Partial magnified drawing of wild type (**O**) and transgenic line 46 (**P**) in N. Bars = 1 cm. (**Q**) statistical analysis of the cotyledon greening/expansion ratio of wild type and transgenic seedlings grown on MS medium containing 0.5 µM ABA. *, P<0.05 and **, P<0.01. WT, wild type; L11, L24 and L46, *GhMPK17* transgenic line 11, 24 and 46.

To investigate the biological function of GhMPK17 in plants, *GhMPK17* gene under the control of CaMV 35 S promoter was introduced into *Arabidopsis* via *Agrobacterium*-mediated DNA transfer (see Methods). Sixty-four transgenic *Arabidopsis* plants were obtained on a selection MS medium with 50 mg/L kanamycin, and the homozygous transgenic progeny lines (T2– T4 generations) were selected through kanamycin-resistance assay and PCR analysis (data not shown). *GhMPK17* expression levels in the transgenic lines were examined by quantitative RT-PCR analysis using gene-specific primers, and three homozygous transgenic lines (L11, L24 and L46) with different expression levels of *GhMPK17* were selected for further analysis. Under normal conditions, *GhMPK17* overexpression transgenic lines were phenotypically indistinguishable from wild type. To study the ABA-tolerance of *GhMPK17* overexpression transgenic *Arabidopsis*, we tested the seed germination rate of wild type and *GhMPK17* overexpression lines on MS medium containing 0, 0.5, 1 or 2 µM ABA. As shown in Fig. 5A, in the absence of exogenous ABA, *GhMPK17* overexpression seeds germinated normally, as did wild type seeds. The seed germination rate of both transgenic lines and wild type reached almost 100% after four days. In the presence of 0.5 µM ABA, seed germination rate of *GhMPK17* overexpression lines was slightly better, compared with that of wild type (Fig. 5B). When sowing on MS medium containing 1 µM ABA, however, seed germination rate of *GhMPK17* overexpression lines was significantly higher than that of wild type. Statistically, wild type had 71.5% seed germination rate, while the transgenic lines L11, L24 and L46 kept 83.5%, 81.5% and 87.5% seed germination rate, respectively (Fig. 5C). Similarly, three transgenic lines still showed significantly higher germination rate (76%, 70% and 86%, respectively) on MS with 2 µM ABA, compared with that (63.5%) of wild type in the same conditions (Fig. 5D).

In addition, *Arabidopsis* seeds were germinated on MS medium under normal conditions for 7 days, and then the seedlings were transferred to grow on MS medium supplemented with 0, 50, 100 and 200 µM ABA for 7 days. Root phenotypes of wild type and transgenic lines were observed and statistically analyzed. As shown in Fig. 5E and 5I, when seedlings grow on MS medium, there was no difference in root elongation between *GhMPK17*-overexpressing lines and wild type. On the contrary, roots of the transgenic plants were much longer than those of wild type in the presence of 50 µM ABA (Fig. 5F). The average root elongations of transgenic seedlings were 20.9 mm (L11), 21.6 mm (L24) and 20.2 mm (L46), respectively, while wild type was 18.1 mm (Fig. 5J). With the increased ABA concentration (100–200 µM), root elongation of both transgenic lines and wild type were severely hindered (Fig. 5G, 5H, 5K and 5L), suggesting that too high ABA concentration may greatly inhibit plant growth.

Furthermore, *GhMPK17* transgenic seedlings on MS medium grew normally, like wild type (Fig. 5M). While sowing on MS medium containing 0.5 µM ABA, cotyledon greening/expansion of both *GhMPK17* transgenic lines and wild type was remarkably inhibited. However, *GhMPK17* transgenic plants showed a higher cotyledon greening/expansion ratio than the wild type (Fig. 5N –5P). Measurement and statistical analysis revealed that there was very significant difference in cotyledon greening/expansion between *GhMPK17* transgenic lines and wild type. 22.5% (L24), 39.8% (L11) and 61.2% (L46) cotyledons of *GhMPK17* transgenic plants turned green/expansion, respectively, whereas only 14.5% of wild type turned green/expansion (Fig. 5Q). These data indicated that overexpression of *GhMPK17* in *Arabidopsis* increased ABA-insensitivity of the transgenic plants, implying that GhMPK17 may be involved in ABA signaling in the transgenic *Arabidopsis*.

### Overexpression of *GhMPK17* in *Arabidopsis* Enhances Plant Tolerance to Salt Stress

**Figure 6:**
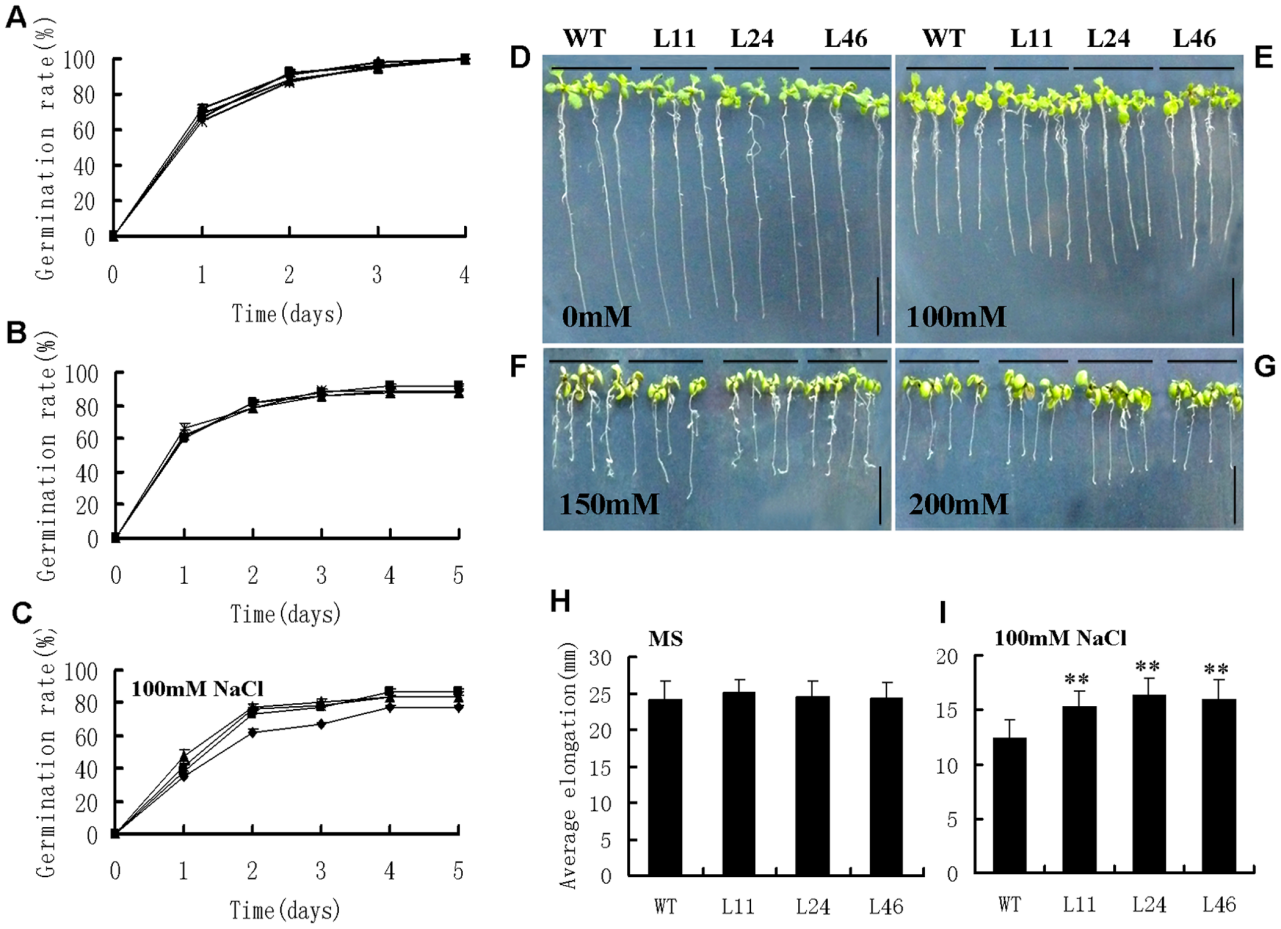
Analysis of seed germination and root elongation of *GhMPK17* overexpression transgenic *Arabidopsis* under NaCl stress. (**A** to **C**) Seed germination rate of *GhMPK17* overexpression lines and wild type on MS medium containing different concentrations of NaCl. (**A**) Seeds germinated on MS medium (control). (**B**) Seeds germinated on MS medium with 50 mM NaCl. (**C**) Seeds germinated on MS medium with 100 mM NaCl. Each curve represents an average of three replicates. (**D** to **G**): Phenotype of wild type and *GhMPK17* overexpression seedlings under NaCl treatment. Seeds were germinated on MS medium under normal condition for 7 days and then the seedlings were transferred onto MS medium supplemented with 0 (**D**), 100 (**E**), 150 (**F**) and 200 (**G**) mM NaCl for 7 days. Each experiment was repeated at least three times with identical results. Bars = 1 cm. (**H** to **I**) Statistical analysis of root elongation of wild type and *GhMPK17* overexpression seedlings grown on MS medium as control (**H**) or MS medium supplemented 100 mM NaCl (**I**). Data were shown from three independent experiments, and bars indicate SDs. **, P<0.01. WT, wild type; L11, L24 and L46, *GhMPK17* transgenic line 11, 24 and 46.

To determine whether the *GhMPK17* was involved in high salinity stress in plants, we tested the seed germination capacity on MS medium with 0, 50 and 100 mM NaCl. On MS medium supplemented 0 (control) and 50 mM NaCl, there was no difference in seed germination rate between wild type and transgenic seedlings (Fig. 6A and 6B). On MS with 100 mM NaCl, however, the *GhMPK17*-overexpressing lines displayed the enhanced tolerance to salt stress, compared with the wild type. The germination rates of transgenic lines were 86.5% (L11), 83% (L24) and 84% (L46), while wild type was 77% (Fig. 6C).

In addition, we also measured and analyzed the root elongation of transgenic plants and wild type when they were cultured on MS medium with different NaCl concentration. On MS medium, root elongation of *GhMPK17*-overexpressing lines was as same as that of wild type (Fig. 6D and 6H). On 100 mM NaCl medium, the root elongation of transgenic plants was significantly longer than that of wild type (Fig. 6B and 6I). With the increased NaCl concentration (150 and 200 mM), root growth of both wild type and transgenic lines was inhibited dramatically (Fig. 6F and G), compared with those under normal conditions. These results indicated that *GhMPK17* enhance plant resistance to salt stress during early plant development.

### Overexpression of *GhMPK17* in *Arabidopsis* Improves Plant Tolerance to Osmotic Stress

**Figure 7:**
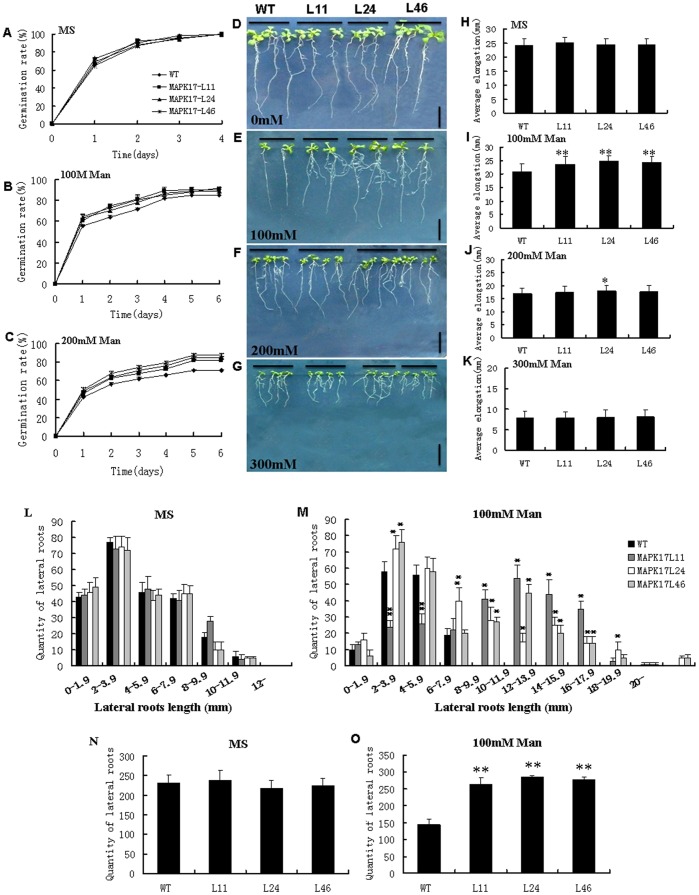
Analysis of seed germination and root elongation of *GhMPK17* overexpression transgenic *Arabidopsis* under mannitol treatment. **(A** to **C**) Seed germination rate of *GhMPK17* overexpression lines and wild type on MS medium containing 0 (**A**), 100 (**B**) and 200 (**C**) mM mannitol. Germination rate (root emergence) was evaluated at indicated time after sowing. (**D** to **G**) Phenotype of wild type and *GhMPK17* overexpression seedlings under mannitol treatment. *Arabidopsis* seeds were germinated on MS medium under normal condition for 7 days and then the seedlings were transferred onto MS medium supplemented with 0 (**D**), 100 (**E**), 200 (**F**) and 300 (**G**) mM mannitol for 7 days. Bars = 1 cm. (**H** to **K**) Average root elongation histogram of wild type and *GhMPK17* overexpression transgenic lines grown on MS medium (**H**), or MS medium supplemented 100 (**I**), 200 (**J**) and 300 (**K**) mM mannitol, respectively. (**L** and **M**) Statistical analysis on the quantity and length of the lateral roots of *GhMPK17* transgenic lines and wild type on MS medium (**L**) or supplemented 100 mM mannitol (**M**). The lateral roots of *GhMPK17* transgenic lines and wild type were divided into several groups according to their length (mm) and the quantity that each group occupied is shown. (**N** and **O**) Histogram of lateral roots quantity of wild type and *GhMPK17* overexpression lines on MS medium (**N**) or supplemented 100 mM mannitol (**O**). Data were shown from three independent experiments, and bars indicate SDs. *, P<0.05 and **, P<0.01. WT, wild type; L11, L24 and L46, *GhMPK17* transgenic line 11, 24 and 46.

To examine if *GhMPK17* responds to osmotic stress in plants, we tested the seed germination capacity on MS medium with 0, 100 and 200 mM mannitol to mimic hyperosmotic condition. In the absence of mannitol, there was little difference in seed germination rate between *GhMPK17* overexpression transgenic lines and wild type (Fig. 7A). In the presence of 100 mM mannitol, the difference in seed germination rate between the transgenic lines and wild type was still not significant (Fig. 7B). Under 200 mM mannitol stress, however, the germination rate of the transgenic seeds was higher than that of wild type. Seed germination percentage of the transgenic lines reached to 82% (L11), 85% (L24) and 87.5% (L46), respectively, while the wild type was 71% on MS medium containing 200 mM mannitol (Fig. 7C).

Additionally, we measured root elongation as an indicator of osmotic stress tolerance. Seeds of both wild type and transgenic lines were germinated on MS medium for 7 days, and then the seedlings were transferred and cultured on MS medium containing 0, 100, 200 and 300 mM mannitol. On MS medium without mannitol (control), there was no difference in root elongation between the transgenic lines and wild type (Fig. 7D and 7H). When treated with 100 mM mannitol, root growth of wild type was inhibited to a greater extent than that of the transgenic seedlings (Fig. 7E). The root elongation of wild type was 20.9 mm, while the transgenic plants reached to 23.6 mm (L11), 25.0 mm (L24) and 24.4 mm (L46), respectively (Fig. 7I). With high concentrations (such as 200 and 300 mM) of mannitol treatment, plant growth was severely inhibited, and no statistical significant differences were detected between transgenic plants (L11 and L46) and wild type (Fig. 7F, 7G, 7J and 7K).

Intriguingly, we observed that the number of lateral roots in the transgenic lines was much more, compared with wild type. Also, the length of lateral roots of the transgenic lines was much longer than that of wild type. After measuring lateral root length, we divided the lateral roots into several groups according to their length (see Methods). As shown in Fig. 7L and 7M, we found that on MS medium, the distribution of the lateral root length of both wild type and *GhMPK17* overexpression lines were almost the same. In each length group, their lateral root quantity did not have obvious distinction. However, when they were treated with 100 mM mannitol, there was significant difference in the number and length of lateral roots between the transgenic lines and wild type. The length of wild type lateral roots was mainly between 2–5.9 mm and not longer than 8 mm, while the length of L11 lateral roots mainly distributed among 8–5.9 mm, and the distribution trend of L24 and L46 was similar to L11. Lateral root length and quantity of the transgenic lines was greater than those of wild type. We separately counted lateral roots of 50 seedlings of each *GhMPK17* transgenic line and wild type. Statistic analysis revealed that there was no difference in root quantity between wild type and transgenic lines on MS medium (Fig. 7N). In contrast, on MS medium supplemented with 100 mM mannitol, the quantity of lateral roots/50 seedlings in the transgenic lines was 263 (L11), 286 (L24) and 277 (L46), respectively, while 143 in wild type. More and longer lateral roots might help the transgenic plants to absorb more water for improving the ability of plant resistance to osmotic stress.

### Overexpression of *GhMPK17* in *Arabidopsis* Reduces H_2_O_2_ Level and Alters Expression of ABA- and Abiotic Stress-related Genes

**Figure 8:**
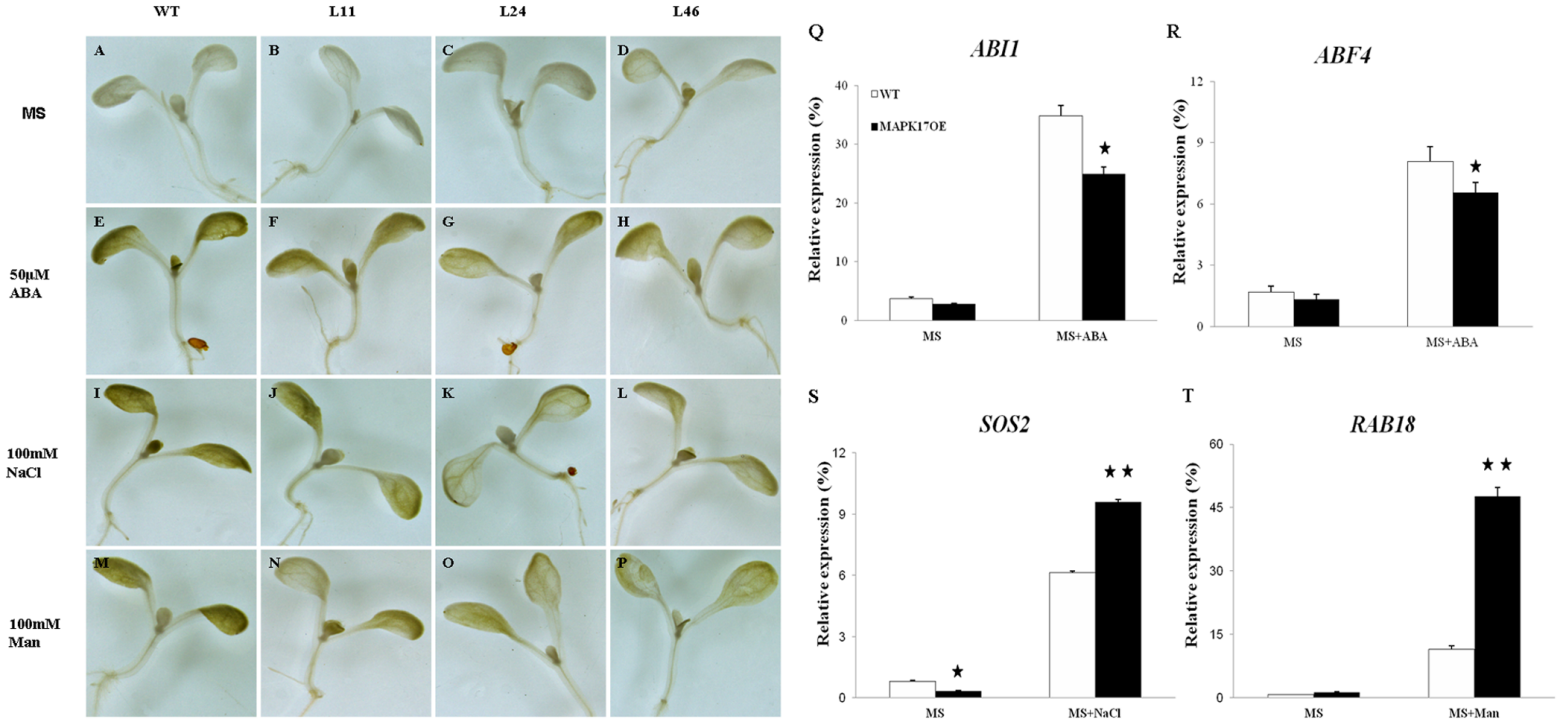
Assays of H_2_O_2_ accumulation and the expression levels of ABA- and abiotic stress-related genes in *GhMPK17* overexpression transgenic *Arabidopsis*. (**A** to **P**) Histochemical assay of H_2_O_2_ accumulation in wild type (**A**, **E**, **I**, **M**) and *GhMPK17* transgenic seedlings (**B** to **D**, **F** to **H**, **J** to **L**, **N** to **P**) on MS medium (**A** to **D**), MS medium supplemented with 50 µM ABA (**E** to **H**), 100 mM NaCl (**I** to **L**) and 100 mM mannitol (**M** to **P**). (**Q** to **T**) Quantitative RT-PCR analysis of expression of ABA-related genes (**Q** and **R**) and abiotic stress-related genes (**S** and **T**) in wild type and *GhMPK17* transgenic seedlings. Total RNA was isolated from two-week-old *Arabidopsis* seedlings grown under normal conditions (MS) and under ABA (MS+ABA), NaCl (MS+NaCl), mannitol (MS+Man) treatments for 6 h. Relative value of the expression of *ABI1*, *ABF4*, *SOS2* and *RAB18* in *Arabidopsis* was shown as percentage of *AtACTIN2* expression activity. Mean values and standard errors (bar) were shown from three independent experiments. *, P<0.05 and **, P<0.01. WT, wild type; L11, L24 and L46, *GhMPK17* overexpression transgenic line 11, 24 and 46; MAPK17OE, *GhMPK17* overexpression transgenic lines.

The accumulation of reactive oxygen species (ROS) is one of the responses of plants under abiotic stress. H_2_O_2_, as one of the ROS, was examined in the transgenic plants and wild type under ABA, NaCl and mannitol treatments. As shown in Fig. 8A–8D, both transgenic seedlings and wild type displayed low levels of H_2_O_2_, and there was little difference in H_2_O_2_ level between the transgenic plants and wild type. On the contrary, the H_2_O_2_ levels of both wild type and transgenic lines were significantly increased on MS medium supplemented with ABA, NaCl and mannitol, but the H_2_O_2_ content in the transgenic seedlings were lower than that in wild type, especially under mannitol treatment (Fig. 8E–8P).

Seed germination, root elongation and H_2_O_2_ level detection indicated an increased ABA-, salt- and osmotic stress-tolerance of the *GhMPK17* overexpressing *Arabidopsis* plants. To investigate the role of H_2_O_2_ in response to abiotic stress, the expression patterns of the ABA and abiotic stress related genes (*ABI1*, *ABF4*, *SOS2* and *RAB18*) were examined in the transgenic seedlings treated with ABA, NaCl and mannitol, using wild type as control. As shown in Fig. 8Q and 8R, the expression levels of *ABI1* and *ABF4* were up-regulated in the transgenic lines and wild type after ABA treatment. However, the expression levels of *ABI1* and *ABF4* in the transgenic seedlings were lower than those in wild type. Similarly, the expression levels of *SOS2* and *RAB18* were dramatically increased in the transgenic lines and wild type under NaCl and mannitol treatments, respectively, and gene expression levels in the transgenic plants were remarkably higher than those in wild type (Fig. 8S and 8T).

## Discussion

Crops in many parts of the world, especially the land of high soil concentration of NaCl, are severely affected by salt stress [30]. Hyperosmotic stress can exacerbate the effects of sodium toxicity in cases where the salt concentration in the soil is particularly high. Several signals are triggered by high salinity and hyperosmotic stresses including ROS formation, PLD-mediated phosphatidic acid production, increment of cytosolic Ca^2+^ concentrations and accumulation of nitric oxide [31]–[34]. Salt and hyperosmotic stresses-induced secondary messengers activate diverse signaling proteins including various members of the MAPK family, which have been implicated in the transduction of the abiotic signals to elicit responses in plants. In *Arabidopsis*, MPK4 and MPK6 are activated by various stresses including salt, drought, cold and wounding [35]. MPK3 is also activated by osmotic stress [36]. MEKK1 mRNA accumulated in response to high salinity [37]. Functional and interaction analysis in yeast revealed protein-protein interactions between MEKK1 and MKK2/MEK1, between MKK2/MEK1 and MPK4, and between MPK4 and MEKK1 [38]. Further studies proved that environmental stress signals are transmitted to at least two MAPK cascades. One is the MPK4 cascade (MEKK1-MEK1/MKK2-MPK4) and the other involves MPK6 and p44MAPK [35]. After identifying MKK2 in a yeast-based screen for activators of MPK4 and MPK6, it was found that MKK2 is activated by salt and cold stress in transient protoplast assays [39]. *mkk2*-null mutant is hypersensitive to cold and salt stresses, and its overexpression transgenic plants are more tolerant to these stresses. MEKK1 can activate MPK4 and MPK6 in an MKK2-dependent manner [40]. Hyperosmotic stress as well as high salinity causes differential activation of the stress-related *Arabidopsis* MPKs [41]. In particular, the activation of MPK6 has been shown to play the central role in the early stage of sodium detoxification, since it rapidly phosphorylates the SOS1 Na^+^/H^+^ antiporter and thereby induces sodium efflux at the expense of cytosolic acidification [42]. A 46-kDa SIMK (salt stress-induced MAPK) in alfalfa was reported to be activated by salt [43]. Yeast-two hybrid assay identified an upstream activator kinase SIMKK that interacts specifically with SIMK and enhanced salt tolerance [44]. Tobacco protoplasts that were exposed to salt and osmotic stresses resulted in enhancement of a 48-kDa kinase, SIPK (salicylic acid-induced protein kinase) [45]. Recently, some salt and hyperosmotic stress-induced MAPKs have been identified in various plants [1]. Likewise, our data revealed that cotton MPK17 is induced in cotton by salt and mannitol treatments, suggesting it may be involved in plant response to salt and osmotic stresses.

Emerging evidence suggests that hormone signaling pathways (such as abscisic acid (ABA), salicylic acid (GA), jasmonic acid (JA), auxin and ethylene), as well as ROS signaling pathway, play crucial roles in the crosstalk between biotic and abiotic stress signaling [46]. These hormones may interact with one another in regulating stress signaling and plant stress tolerance. Especially, ABA is an important hormone that mediates the adaptation of plants to unfavorable environmental conditions including salinity, cold, drought and pathogen attacks. Treating plants with ABA induces the transcriptional regulation, protein accumulation and stability, kinase activity of several components of distinct MAPK signaling cascades in many plant species, indicating the involvement of MAP kinase cascades in ABA transduction pathway [47], [48]. In *Arabidopsis*, treatment with ABA induced the transcriptional regulation of *MPK3*, *MPK5*, *MPK7*, *MPK18*, *MPK20*, *MKK9*, *MAPKKK1*, *MAPKKK10*, *MAPKKK14*, *MAPKKK15*, *MAPKKK16*, *MAPKKK17*, *MAPKKK18*, *MAPKKK19*, *Raf6*, *Raf12*, and *Raf35* [49]. In other plant species, several MAPKs have also been reported to be transcriptionally activated by ABA. In rice, the ABA-inducible *OsMAPK5* was shown to act as a positive regulator in abiotic stress tolerance and as a negative regulator of PR gene expression and broad-spectrum disease resistance [13]. In maize, *ZmMKK3* was involved in osmotic stress and ABA responses [50]. ABA has also been shown to induce transcription of MAPK genes in other plant species [51]–[53]. Three isoforms of catalase, CAT1, CAT2 and CAT3, have been discovered in *Arabidopsis* [54]. It was assumed that ABA-induced expression of *CAT1* was regulated by *Arabidopsis* MKK1 in response to H_2_O_2_ signaling [55]. Another component that was found to be involved in this signaling pathway is MPK6. It is therefore likely that the MKK1/MPK6 module is an important component of the ABA-dependent signaling pathway that is responsible for H_2_O_2_ production and stress responses [47]. Similarly, we found that *GhMPK17* expression was up-regulated in cotton by ABA, and overexpression of *GhMPK17* in *Arabidopsis* increased ABA-tolerance of the transgenic plants, implying that GhMPK17 may be involved in ABA signaling during cotton development.

In the present study, we identified a MAPK protein (GhMPK17) of group D in cotton. GhMPK17 contains a TDY activation motif in its T-loop, the extended C-terminal region and the lack of the CD domain, compared with the TEY subtypes of MAPKs. Intriguingly, *GhMPK17* was up-regulated in cotton seedlings under NaCl, mannitol and ABA treatments. Overexpression of *GhMPK17* in *Arabidopsis* enhanced plant tolerance to salt and osmotic stresses, and altered H_2_O_2_ level and expression of abiotic stress-related genes in the transgenic plants. These data suggested that GhMPK17 may be involved in cotton response and tolerance to salt and osmotic stresses in ABA-dependant manner.
